# Alzheimer's Disease: A Pathogenetic Autoimmune Disorder Caused by Herpes Simplex in a Gene-Dependent Manner

**DOI:** 10.4061/2010/140539

**Published:** 2010-12-29

**Authors:** C. J. Carter

**Affiliations:** Polygenic Pathways, Flat 4, 20 Upper Maze Hill, Saint Leonard's on Sea, East Sussex TN38 OLG, UK

## Abstract

Herpes simplex is implicated in Alzheimer's disease and viral infection produces Alzheimer's disease like pathology in mice. The virus expresses proteins containing short contiguous amino acid stretches (5–9aa “vatches” = viralmatches) homologous to APOE4, clusterin, PICALM, and complement receptor 1, and to over 100 other gene products relevant to Alzheimer's disease, which are also homologous to proteins expressed by other pathogens implicated in Alzheimer's disease. Such homology, reiterated at the DNA level, suggests that gene association studies have been tracking infection, as well as identifying key genes, demonstrating a role for pathogens as causative agents. Vatches may interfere with the function of their human counterparts, acting as dummy ligands, decoy receptors, or via interactome interference. They are often immunogenic, and antibodies generated in response to infection may target their human counterparts, producing protein knockdown, or generating autoimmune responses that may kill the neurones in which the human homologue resides, a scenario supported by immune activation in Alzheimer's disease. These data may classify Alzheimer's disease as an autoimmune disorder created by pathogen mimicry of key Alzheimer's disease-related proteins. It may well be prevented by vaccination and regular pathogen detection and elimination, and perhaps stemmed by immunosuppression or antibody adsorption-related therapies.

## 1. Introduction

Herpes simplex infection (HSV-1) has been shown to be a risk factor in Alzheimer's disease; acting in synergy with possession of the APOE4 allele HSV-1 infection in mice or neuroblastoma cells increases beta-amyloid deposition and phosphorylation of the microtubule protein *tau* [1–5]. Viral infection in mice also results in hippocampal and entorhinal cortex neuronal degeneration, brain shrinkage, and memory loss, all as found in Alzheimer's disease [6]. A recent study has also shown that anti-HSV-1 immunoglobulin M seropositivity, a marker of primary viral infection or reactivation, in a cohort of healthy patients, was significantly associated with the subsequent development of Alzheimer's disease. Anti-HSV-1 IgG, a marker of lifelong infection, showed no association with subsequent Alzheimer's disease development [7]. All of these factors support a viral influence on the development of Alzheimer's disease. As shown below, proteins expressed by HSV-1 are homologous to all of the protein products of the major susceptibility gene in Alzheimer's disease (APOE, clusterin, complement receptor 1, and PICALM) as well as to APP and *tau* and over 100 others implicated in genetic association studies. This suggests that Alzheimer's disease is a “pathogenetic” disorder caused by HSV-1 (and other infections) that mimic these key susceptibility targets.

## 2. Methods

The Human herpesvirus 1 genome (NC_001798) was screened against the human proteome using the NCBI BLAST server with and without the Entrez Query filters (“Alzheimer” or “cholesterol”) [8]. Each BLAST returns a large list of human proteins, many of which display homology to several different HSV-1 proteins. A Tag cloud generator was used to quantify these different interactions http://www.tagcloud-generator.com/index.php. This generates tags whose font size is proportional to the number of viral protein hits per human protein. The tag size scale was set from 1 to 20. Antigenicity (B cell epitope prediction) was predicted using the BepiPred server [9] at http://www.cbs.dtu.dk/services/BepiPred/ and T cell epitopes predicted using the Immune epitope database resource at http://tools.immuneepitope.org/main/html/tcell_tools.html  [10]. The immunogenicity index for individual amino acids is shown in Table 1. References for genetic association studies can be found at http://www.polygenicpathways.co.uk/alzpolys.html. References for herpes simplex host viral interactions can be found in a database at http://www.polygenicpathways.co.uk/herpeshost.html. Protein kinases phosphorylating the microtubule protein *tau* were identified from the Kinasource database at http://www.kinasource.co.uk/Database/welcomePage.php and from the material available at the ENTREZ gene interaction section for *tau* (MAPT). 

 Because of the large volume of data generated by the BLASTs, raw BLAST data have been made available at http://www.polygenicpathways.co.uk/Alzheimer.htm. This survey is restricted to the herpes simplex virus, HSV-1, but similar data were obtained for other viral or pathogen species implicated in Alzheimer's disease, where similar conclusions apply. These BLAST files and a summary of the results are available on the PolygenicPathways website at http://www.polygenicpathways.co.uk/BLASTS.htm.

## 3. Results

The results of the HSV-1 BLASTS, sized according to the number of viral hits per protein, using the filter “Alzheimer,” are shown in Table 2. Over a hundred human gene products, including all of the major Alzheimer's disease susceptibility gene products (APOE4, clusterin, complement receptor 1, and PICALM) and most of many other diverse genes that have been implicated in Alzheimer's disease in genetic association studies contain intraprotein sequences that are identical to those within herpes simplex viral proteins. The alignment with complement receptor 1 (CR1) has functional consequences, as glycoprotein C of the virus acts as a CR1 mimic, binding to other complement components (C3 and its derivatives) blocking the complement cascades and preventing formation of the membrane attack complex [12, 13]. This nicely illustrates one of the functional consequences of this type of mimicry. 

The type of viral homology for various different protein classes is shown in Table 3. These classes include products involved in APP signalling and processing (BACE1 and 2 and gamma-secretase components), cholesterol and lipoprotein function, *tau* function, inflammation, and oxidative stress, all of which are key processes disrupted in the Alzheimer's disease brain.

Using the filter “cholesterol,” a number of cholesterol and lipoprotein-related proteins again contain numerous sequences corresponding to those found in herpes viral proteins. This group of proteins play an important role in Alzheimer's disease pathophysiology [14–17].

The unfiltered BLAST returns the human proteins with the greatest homology to viral proteins and showed that herpes simplex viral proteins are highly homologous to a series of family members of diverse protein kinases. Several of these are known to phosphorylate the microtubule protein *tau*, an effect that is observed following HSV-1 infection [5]. The homology is such as to suggest that such phosphorylation may be accomplished by the viral proteins themselves, as well as by human protein kinases (Table 4).

This type of mimicry is by no means restricted to the herpes simplex virus as APOE4, clusterin, complement receptor 1, and PICALM are homologous to proteins from a diverse array of phages and viruses including phages that affect commensal bacteria, the influenza virus, and the HHV-6 virus which has a seroprevalence approaching 100% [18] (Table 5). Because of the universality of the phenomenon of viral matches within the human proteome, most proteins will be homologous to proteins from specific subsets of viruses. Viruses and other pathogens expressing proteins with homology to key susceptibility gene products might however be considered as important potential environmental risk factors. For the major Alzheimer's disease gene candidates, several herpes species other than HSV-1 (HSV-2, 3, 6, 6B, and 8) fall into this category (Table 5). 

The tables in supplementary data on the website http://www.polygenicpathways.co.uk/alzheimer.htm show that numerous Alzheimer's disease susceptibility gene products are also homologous to proteins expressed by other pathogen risk factors in Alzheimer's disease, including Chlamydia pneumonia, which has recently been detected in the Alzheimer's disease brain [19].

 Cryptococcus neoformans, Helicobacter pylori, Porphyromonas gingivalis (one cause of the gum disease that is a risk factor in Alzheimer's disease [20]), Borrelia Burgdorferi, [21], Human herpesvirus 6, and Human herpesvirus 5 (Cytomegalovirus) [22]. 

Cryptococcus neoformans infection has been shown to be associated with a rare but curable form of dementia in two separate studies, where both patients had been consigned to healthcare for 3 years, with a diagnosis of Alzheimer's disease. Both recovered normal function following antifungal treatment [23, 24]. Heliocobacter pylori eradication has also been reported to improve cognitive function in Alzheimer's disease [25]. 

The protein sequences highlighted in grey in Table 3 contain strings of herpes simplex proteins that have been shown to bind to several interactome partners of *tau* [11] (see http://www.polygenicpathways.co.uk/herpeshost.html) and are those most likely to form epitopes that cross-react with their human counterparts (Table 1). These include APOE4, complement receptor 1, clusterin, insulin degrading enzyme, the APP homologue, APLP2, the APP binding protein APBBI1P, the collagen amyloid plaque component CLAC, synuclein, and the foetal Alzheimer antigen, ALZ50. Tau appears to be highly antigenic (Table 2).

This antigenicity was further studied for the two key proteins in Alzheimer's disease, beta-amyloid and *tau*, and the predicted immune epitopes compared with the HSV-1 viral proteins aligning within these various regions (Figures 2 and 3).

## 4. Vatches within Beta-Amyloid and the Microtubule Protein *tau*

Vatches (= viralmatches) are short contiguous amino acid stretches that are identical in viral and human proteins [26, 27]. There are several million within the human proteome, derived from evolutionary descent and from the insertion of multiple viruses into the human genome over millions of years. This type of insertion is not restricted to retroviruses, as herpes viruses, hepatitis viruses, influenza and the common cold virus, the coronavirus, and the papillomavirus, among others, have all been inserted into different genomic regions or are homologous to the encoded protein products. This has occurred on several occasions during evolutionary time, and these reinsertions appear to be responsible for the creation of gene families (see http://www.polygenicpathways.co.uk/blasts.htm), where over 2 million such alignments are available for multiple viral species. In effect, the entire human genome appears to be composed of viral DNA. For example, the coverage of human chromosome 10 is complete, with 119,867 human/viral DNA matches.

**Figure 1:**
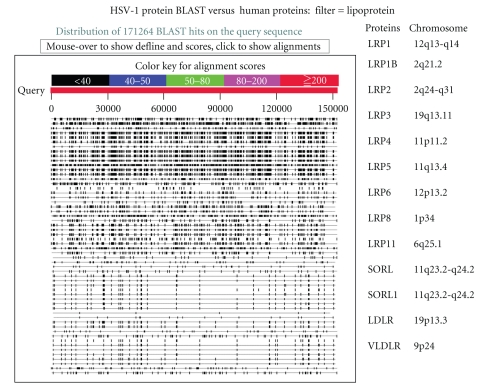
The BLAST result for HSV-1 proteins (translated viral genome versus human proteins) using the filter “lipoprotein.” The repetitive patterns in the pictogram reflect homology with a number of different lipoprotein receptors located on different chromosomes, as shown in the table.

A single HSV-1 vatch, translated back to DNA, is identical to DNA in 103 different genomic regions covering several human chromosomes. This phenomenon is likely responsible for the creation of gene families, and the HSV-1 virus appears to have been partly responsible for the creation of lipoprotein receptor families (Figure 1), and of numerous kinases within a number of different families (see above and Table 2). Over millions of years, these DNA inserts have been extensively shuffled by recombination, but millions of consecutive sequences are retained that encode for the viral matching protein components. 

**Figure 2:**
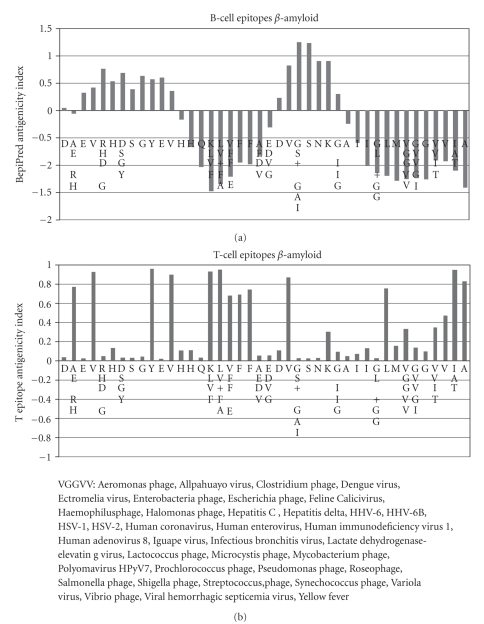
The B cell and T cell immunogenicity profile for the beta-amyloid peptide. According to the servers, antigenicity values of >0.35 (B cell) or 0.5 (T cell) are considered immunogenic. The sequences of herpes simplex viral proteins that align with beta-amyloid are shown. Space: non-identical amino acid; +: conserved amino acid with similar physicochemical properties. Viruses and phages containing the VGGVV sequence, which has been used as an epitope to label beta-amyloid in Alzheimer's disease, are also shown.

**Figure 3:**
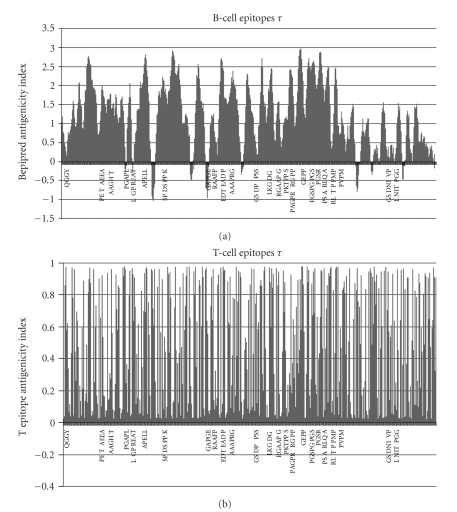
The B cell and T cell immunogenicity profile for the *tau* protein. The sequences of herpes simplex viral proteins that align with *tau* are shown. Space: non-identical amino acid; +: conserved amino acid with similar physicochemical properties.

Some of the vatches within beta-amyloid and *tau* are illustrated in Figures 2 and 3 which also demonstrates the B cell and T cell antigenicity of these proteins. As can be seen, there are numerous HSV-1 vatches within both proteins, many of which correspond to highly antigenic regions of APP or *tau*, and therefore also of the HSV-1 proteins.

In addition to the herpes simplex virus, a large number of other viruses express proteins containing a VGGVV sequence that is identical to that of a C-terminus peptide within beta-amyloid. Although not the most immunogenic of sequences, this epitope has been used to label beta-amyloid in Alzheimer's disease brain [28] (Figure 2).

## 5. HSV-1 Proteins Bind to the Interaction Partners of *tau*

Because HSV-1 proteins are homologous to portions of the *tau* protein, one might expect the viral proteins to interfere with *tau* binding partners. This is indeed the case, as diverse herpes simplex viral proteins have been shown to bind to several of the interactome partners of *tau* (Table 6).

## 6. Discussion

Almost without exception, the genes encoding the proteins that match HSV-1 sequences (using the filter “Alzheimer”) have been reported as genetic risk factors in Alzheimer's disease (see http://www.polygenicpathways.co.uk/alzpolys.html) suggesting that such studies have been tracking HSV-1 (and other) infections over the years and inadvertently demonstrating that HSV-1 causes Alzheimer's disease. This in no way detracts from the importance of these studies, but reflects a phenomenon that is probably common to most diseases. Because of our likely evolutionary descent from viruses, first opined by J.B.S. Haldane and Francois D'Herelle almost a century ago [29, 30], our genomes contain traces of this descent which are transcribed into these short contiguous amino acid stretches (vatches) that exactly match many of the proteins in the current virome. Repeated viral insertions also add several genes to the human genome at once, a phenomenon that is likely responsible for evolutionary jumps, as suggested by others [31]. The idea that higher forms of life originated from viruses, although contentious, is supported by the fact that the entire human genome appears to be comprised of viral DNA. For example a BLAST of human chromosome 10 against all viral genomes (DNA versus DNA) returned 119,867 hits, covering the entire chromosome, with no gaps, in both inter- and intragenic regions (see http://www.polygenicpathways.co.uk/viralimages.htm). Similar results were obtained for other chromosomes. Our genomes and polymorphisms thus determine which vatches we possess, which viruses pose the threat, and which viral-related disease we are likely to develop. Whether we develop the disease in question will depend on our encounters with the virus, whether we are vaccinated, and no doubt on our HLA-antigens and immune background related to the elimination of self-antibodies soon after birth.

This phenomenon appears to be universal, as vatches have been found in the XMRV virus, relating to human proteins involved in mitochondrial respiration and prostate cancer, in the Epstein-Barr virus, which matches multiple sclerosis autoantigens [27], in the AIDS virus which targets vatches in over 50 components of the human immune network, in the papillomavirus which targets cervical cancer oncogenes, and in the HSV-2 virus which targets schizophrenia susceptibility gene products (see http://www.polygenicpathways.co.uk/BLASTS.htm). It is even relevant to human genetic diseases as the polyglutamine repeats observed in Huntington's disease and spinocerebellar ataxias align with very common viruses (the ubiquitous HHV-6) while the cystic fibrosis mutant aligns with pseudomonas and staphylococcal phages, whose bacterial hosts have been found to shorten the lifespan of these patients. The London mutation in Alzheimer's disease converts the surrounding peptide to a vatch that is homologous to proteins from the rhinoviruses that cause the common cold [26, 27, 32, 33]. Every human protein so far screened by the author, without a single exception, displays this type of homology to particular but specific sets of virus for each protein. Similarly all viruses so far screened (~30) express proteins with homology to a large but specific subset of human proteins. 

These viral homologues may interfere with Alzheimer's disease pathological pathways in a number of ways. Firstly, as demonstrated by the complement receptor 1 HSV-1 viral mimic, the viral protein can substitute for its human counterpart, presumably diverting its function towards different compartments. Secondly, as they are clearly able to substitute for their human counterparts, they are likely to interfere with their protein/protein networks (interactome). This was clearly demonstrated for *tau*, where herpes simplex virus proteins do indeed bind to *tau* binding partners. 

As many of these matching sequences are highly immunogenic, antibodies to the virus may also target the human homologue, in effect producing a protein knockdown and reproducing the effects, but on a massive scale, seen in various Alzheimer's disease-related knockout mice [34–39]. Such immunogenic viral proteins may also generate antibodies capable of mounting an immune attack against their human counterparts, killing the cells in which they reside by immune and inflammatory mechanisms, and by complement-related lysis (see below).

## 7. The Dangers of Autoimmunity

The immunogenic profile of some of these homologues may also be responsible for the neurodegeneration and pathological features observed in Alzheimer's disease. Antibodies to the human proteins may result in immune, inflammation, and complement pathway activation, killing the cells in which the human homologue resides. There is a great deal of evidence supporting autoimmune attack in the Alzheimer's disease brain.

A number of immune-system-related proteins are found in amyloid plaques or neurofibrillary tangles. Interleukin 1 alpha, interleukin 6, and tumour necrosis factor are all been localised within plaques, and acute phase proteins involved in inflammation, such as amyloid P, alpha-1 antichymotrypsin, and C-reactive protein are also plaque components while immunoglobulin G is located in the plaque corona [14, 40–42]. Large increases in IgG levels have been recorded in the brain parenchyma, in apoptotic dying neurones, and in cerebral blood vessels in the Alzheimer's disease brain [43]. Complement component C3 is found in Alzheimer's disease amyloid plaques along with complement C4 [44]. Complement components Clq, C3d, and C4d are present in plaques, dystrophic neuritis, and neurofibrillary tangles [45]. 

The membrane attack complex (MAC), composed of complement proteins C5 to C9, forms a channel that is inserted into the membranes of pathogens, destroying them by lysis. These components cannot be detected in temporal cortex amyloid plaques in Alzheimer's disease [41, 44, 46]. However the MAC complex is present in dystrophic neurites and neurofibrillary tangles [45], and others have detected this complex in neuritic plaques and tangles, along with deposition of C1q, C3, and clusterin [47]. The membrane attack complex has also been detected in the neuronal cytoplasm in AD brains and associated with neurofibrillary tangles and lysosomes [46].The presence of the MAC complex in neurones might suggest that neuronal lysis by the MAC complex could contribute to neuronal cell death [45].

The microtubule protein *tau* was one of the more antigenic proteins revealed in this survey and one with numerous matches to herpes viral proteins that would be equally immunogenic. Immunisation with *tau* in mice produces tauopathy, neurofibrillary tangles, axonal damage, and gliosis [48] demonstrating the dangers of autoimmunity in a manner directly relevant to Alzheimer's disease. 

Beta-amyloid autoantibodies are common in the ageing population and in Alzheimer's disease and may be related to herpes simplex and numerous other viruses or phage proteins that exactly vatch a VGGVV C-terminal sequence in beta-amyloid that is immunogenic. The epitope for this sequence labels beta-amyloid in the Alzheimer's brain [28]. This pentapeptide is, *per se*, fibrillogenic [49]. This is a characteristic of both beta-amyloid and of HSV-1 glycoprotein B peptide fragments containing this sequence. The viral glycoprotein B fragments form thioflavin T positive fibrils which accelerate beta-amyloid fibril formation and are neurotoxic in cell culture [50]. Other stretches of beta-amyloid are homologous to a diverse set of viral, bacterial, fungal, and allergenic proteins, likely providing the source of the autoantibodies in the ageing population [32].

Antibodies to beta-amyloid have been suggested as a therapeutic option in Alzheimer's disease. The potential use of beta-amyloid antibodies is based on their ability to reduce plaque burden and neurite dystrophy in APP transgenic mice [51]. Several studies have demonstrated that beta-amyloid antibodies reduce plaque burden in APP transgenic models and that they can also improve cognitive performance [52]. However amyloid antibodies extracted from the serum of old APP transgenic mice potentiate the toxicity of beta-amyloid, and Alzheimer's disease patients display an enhanced immune response to the peptide [53]. Again in transgenic mice, different immune backgrounds can influence the type of immune responses elicited by beta-amyloid. For example, B and T cell responses to beta-amyloid can be modified in HLA-DR3, -DR4, -DQ6, or -DQ8 transgenic mice [54]. HLA-antigen diversity in Man is also likely to determine the outcome of beta-amyloid/antibody interactions. A large number of Alzheimer's disease susceptibility gene candidates, including clusterin and complement receptor 1, as well as diverse interleukins and other cytokines, C reactive protein, HLA-antigens, Fc epsilon and Toll receptors, and the viral-activated kinase PKR, are intimately concerned with pathogen defence and or the immune system, supporting a genetic contribution to the immune pathogenesis of Alzheimer's disease (see http://www.polygenicpathways.co.uk/alzpolys.html.).

Beta-amyloid vaccination in Alzheimer's disease (against Abeta_1-42_) has so far not been successful and sadly resulted in meningoencephalitis and the death of a patient [55]. While certain beta-amyloid antibodies may reduce plaque burden, there is an evident risk that they may also trigger an autoimmune response, potentially killing beta-amyloid containing neurones. Catalytic autoantibodies are less able to form stable immune complexes and likely represent the safest way forward in this area [56, 57]. Given the homology of beta-amyloid to so many viruses and the potential dangers of autoimmunity, as well as the clearly toxic effects of *tau* immunisation, the pursuit of clinical trials with beta-amyloid antibodies, with the exception of catalytic forms, must surely be questioned. 

## 8. Conclusions

Alzheimer's disease proteins encoded by all of the major genetic players in Alzheimer's disease and many other relevant proteins are homologous to proteins from the herpes simplex virus, confirming the implication of this virus as a causative agent in this disease [48, 50, 58–70]. Because of homology to other viruses and pathogens, these too may be implicated. These include HHV-6, the cytomegalovirus, Borrelia, Burgdorferi, Chlamydia Pneumoniae, Helicobacter pylori, Cryptococcus neoformans and bacteria promoting gum disease, such as P. Gingivalis, all of which also express proteins homologous to the products of numerous Alzheimer's disease susceptibility genes (see http://www.polygenicpathways.co.uk/Alzheimer.htm).

No vaccine against HSV-1 exists, but in the long term, may perhaps be able to prevent Alzheimer's disease, although the potential dangers of vaccine-related autoimmunity evidently need to be addressed. Interestingly, cancer-causing viruses including the Epstein-Barr-virus, hepatitis b, and the papillomavirus align with the peptide stretch within beta-amyloid [32] that is cleaved by the beneficial catalytic autoantibodies to beta-amyloid [56, 57]. Cancer is inversely associated with the risk of developing Alzheimer's disease [71, 72]. As a vaccine to the human papillomavirus already exists to prevent cervical cancer [73], it may well have a role to play in the prevention or therapy of Alzheimer's disease, again with due regard to the problem of vaccine-related autoimmunity. Alternatively, immunisation with this beneficial region of the beta-amyloid peptide might be considered as a viable therapeutic option. 

 Many of the toxic effects of HSV-1 infection are likely to be related to autoimmunity, caused by antibodies to the viral proteins that also target their human counterparts. In this case, it is possible that immunosuppressant therapy may be of benefit in Alzheimer's disease patients and also that aggressive antiviral therapy should be pursued. Immunoadsorption of *tau* and beta-amyloid antibodies, a technique used to good effect in certain patients with myasthenia gravis (characterised by autoantibodies to nicotinic receptors) [74] may also be of benefit. As other pathogens may also demonstrate this type of mimicry, detailed and regular pathogen screens in the ageing population and in the early stages of Alzheimer's patients may also be of use. 

Alzheimer's disease thus appears to be one, probably of many, “pathogenetic” diseases, caused by viruses and other pathogens, but dependent on our genes, which dictate the protein sequences that match those in particular subsets of pathogen proteins. There are almost 3,000 viral genomes in the NCBI database, probably reflecting but a small proportion of those existing on the planet. In addition, as viruses regularly mutate with replication there are likely to be multiple strains of HSV-1 (and other viruses), only one of which is recorded in the NCBI database. Nevertheless, with current bioinformatics techniques, it should be possible to rapidly identify all vatches in the human proteome, to match them to particular viruses (and other pathogens, Bacteria, fungi, yeast, parasites, etc.), and to pair these with diverse human diseases. Our understanding of this universal phenomenon could radically change the face of therapy in a variety of human conditions. 

## Figures and Tables

**Table 1:** The antigenicity index (B cell epitope) for single amino acids defined by the BepiPred server. The top 6 scoring amino acids are highlighted in grey in the various tables.

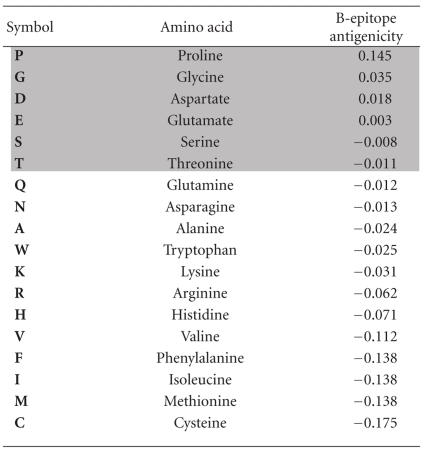

**Table 2:** Human proteins with homology to HSV-1 proteins: The size of symbol (HUGO Nomenclature approved gene symbols) is proportional to the number of viral proteins displaying homology to the gene product. Filter “Alzheimer”: all of the genes encoding for these proteins with the exception of those with the strikethrough have been implicated in Alzheimer's disease in genetic association studies. Filter “cholesterol”: genes encoding for proteins products in dashed boxes have been implicated in Alzheimer's disease in genetic association studies. No Filter: HSV-1 proteins are most homologous to diverse families of kinases: Those boxed have been shown to phosphorylate the microtubule protein *tau* (Data from Kinasource and from NCBI Interactions section for the MAPT gene (*tau*)).

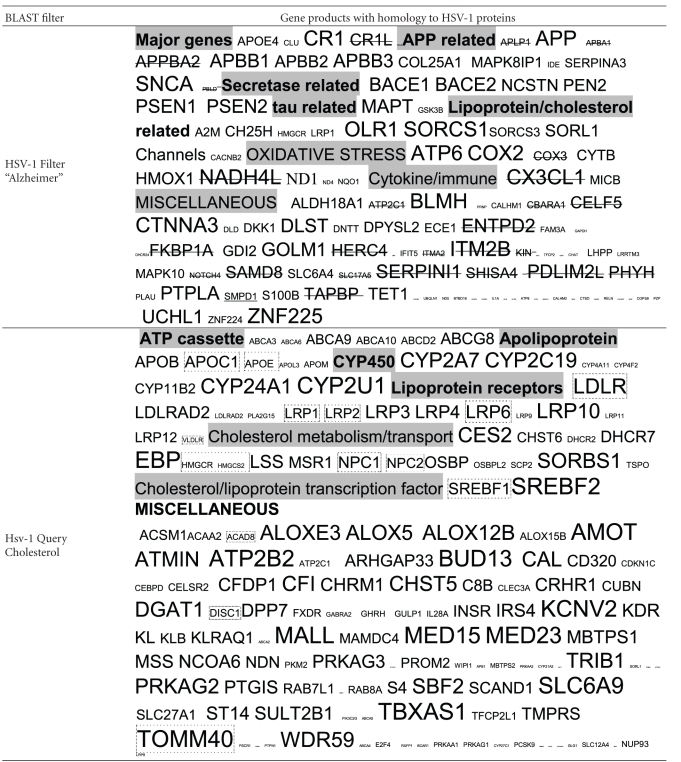 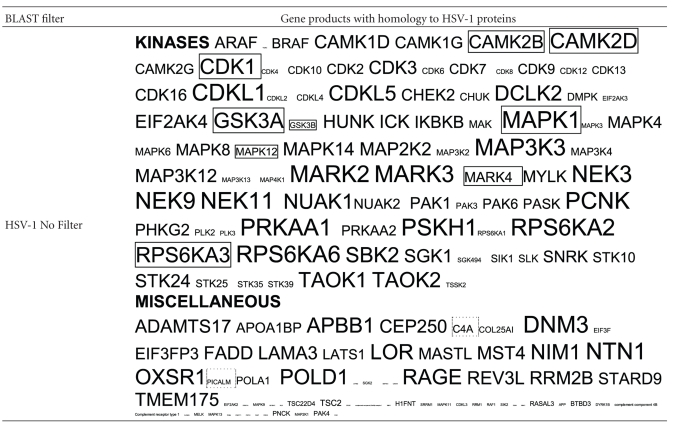

**Table 3:** Major susceptibility gene products and members of other key signalling networks in Alzheimer's disease (Sbjct) aligning with the translated HSV-1 genome (Query). The 6 amino acids with the highest B cell antigenicity index are highlighted in grey (see Table 1). Spaces denote a nonidentical amino acid; dashes represent gaps and + = conserved amino acid (similar physicochemical properties).

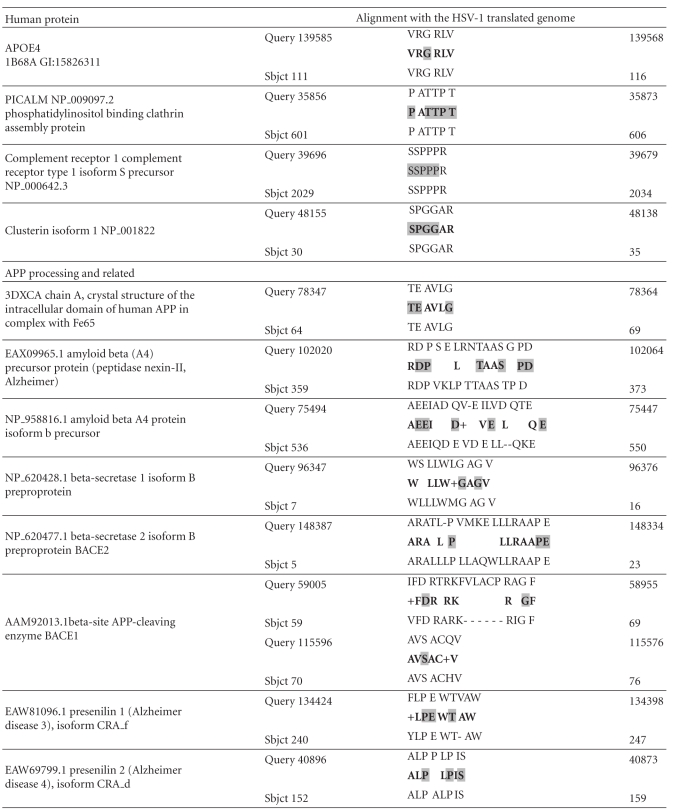 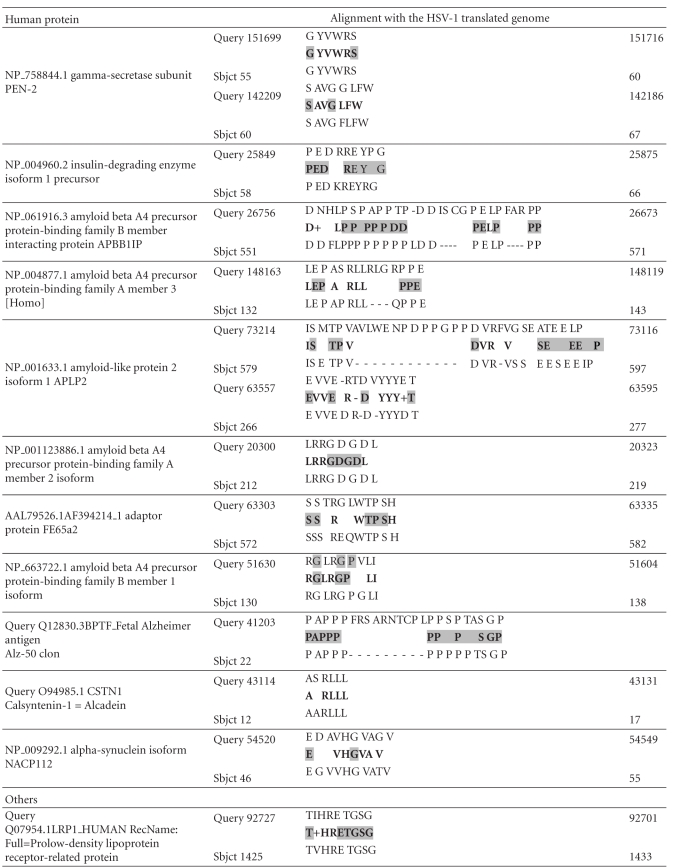 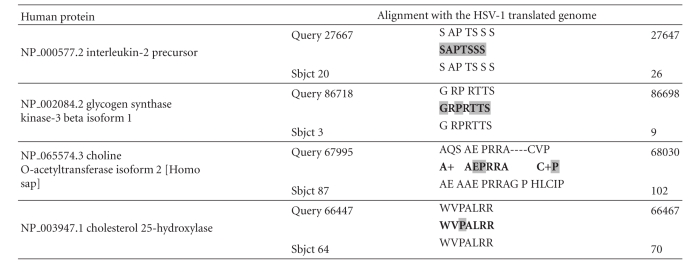

**Table 4:** Alignment of the HSV-1 translated genome (Query) with 3 protein kinases known to phosphorylate *tau* (Sbjct). Glycogen synthase kinase GSK3A aligns with the same amino acids as GSK3B. CAMK2B: calcium/calmodulin-dependent protein kinase II beta. MAPK1: mitogen-activated protein kinase 1 (erk2).

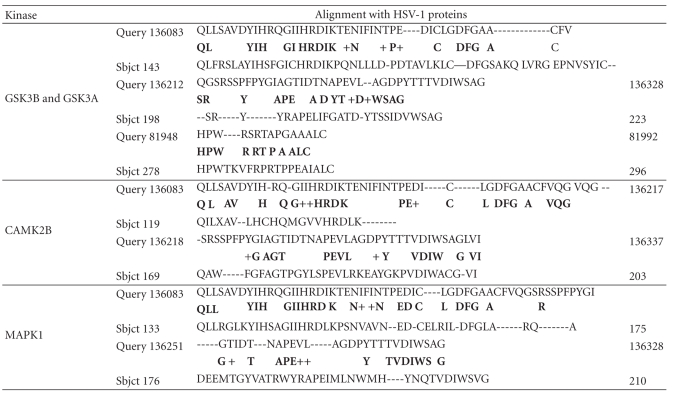

**Table 5:** Other viruses expressing homologous proteins for the four major Alzheimer's disease susceptibility gene products.

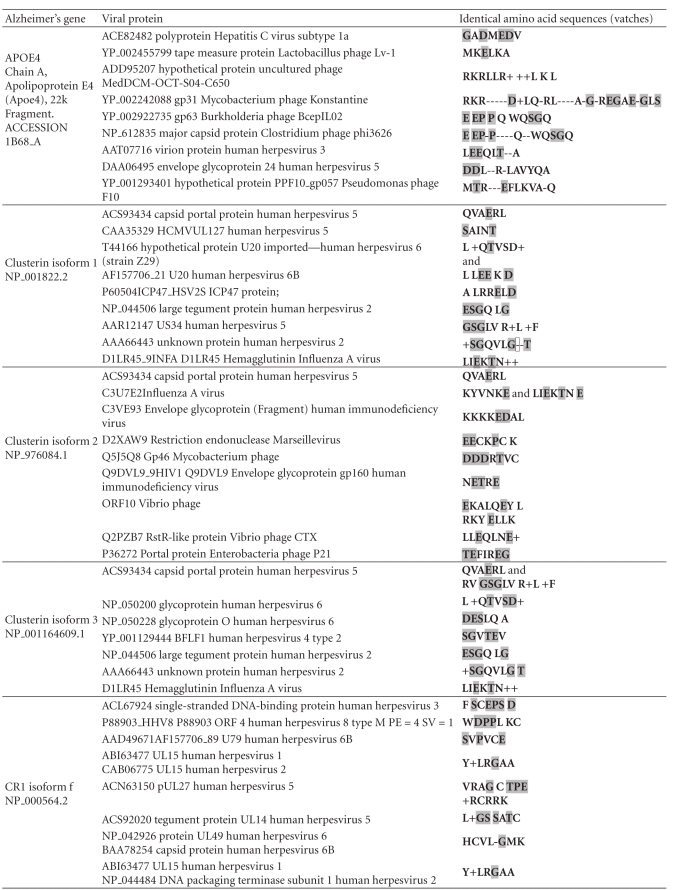 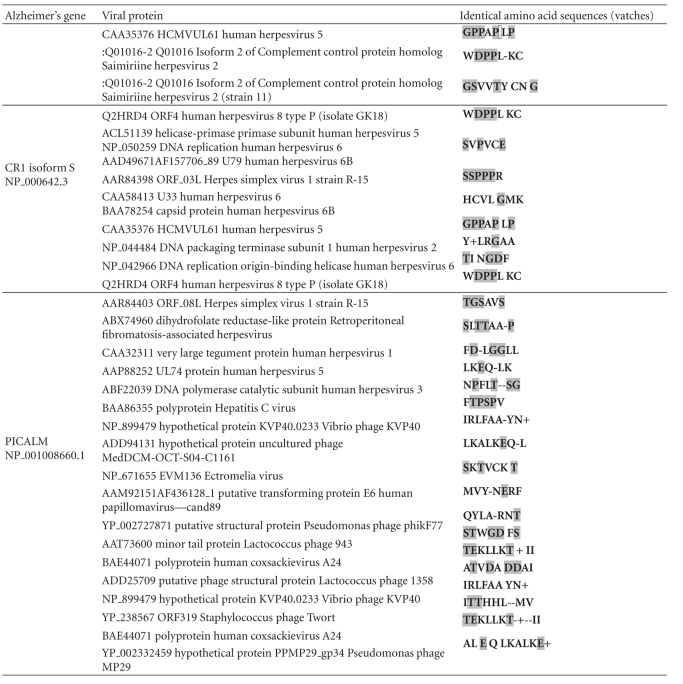

**Table 6:** The binding partners of *tau* (from the interaction section of NCBI gene) and their interaction with herpes simplex proteins (from the Wikigenes database) [11]; https://www.wikigenes.org/e/art/e/61.html.

Gene symbol	Name	Interaction with HSV-1 proteins
**AATF**	Apoptosis antagonizing transcription factor	—
**ABL1**	V-abl Abelson murine leukemia viral oncogene homolog 1	—
**ACTB**	Actin, beta	Virion component
**APOE**	Apolipoprotein E	Binds to glycoprotein B
**BAG1**	BCL2-associated athanogene	—
**CALM1**	Calmodulin 1 (phosphorylase kinase, delta)	Phosphorylated by ICP10
**CAMK2A**	Calcium/calmodulin-dependent protein kinase (CaM kinase) II alpha	—
**CASP1**	Caspase 1, apoptosis-related cysteine peptidase (interleukin 1, beta, convertase)	—
**CASP3**	Caspase 3, apoptosis-related cysteine peptidase	US3 phosphorylates procaspase 3
**CASP6**	Caspase 6, apoptosis-related cysteine peptidase	—
**CASP7**	Caspase 7, apoptosis-related cysteine peptidase	Activated during HSV-1 mediated apoptosis
**CASP8**	Caspase 8, apoptosis-related cysteine peptidase	Activity inhibited by LAT latency transcript
**CDK1**	Cyclin-dependent kinase 1	—
**CDK5**	Cyclin-dependent kinase 5	—
**FLJ10357**	Hypothetical protein FLJ10357	—
**FYN**	FYN oncogene related to SRC, FGR, YES	—
**GSK3A**	Glycogen synthase kinase 3 alpha	—
**GSK3B**	Glycogen synthase kinase 3 beta	Activated by HSV-1 infection
**HSPA8**	Heat shock 70 kDa protein 8	Recruited to nuclear domains following infection: ICP0 dependent
**MAPK12**	Mitogen-activated protein kinase 12	—
**MAPT**	Microtubule-associated protein *tau *	Phosphorylated by viral infection via GSK3B and PRKACA
**MARK1**	MAP/microtubule affinity-regulating kinase 1	—
**MARK4**	MAP/microtubule affinity-regulating kinase 4	—
**OGT**	O-linked N-acetylglucosamine (GlcNAc) transferase (UDP-N-acetylglucosamine:polypeptide-N-acetylglucosaminyl transferase)	—
**PARK2**	Parkinson disease (autosomal recessive, juvenile) 2, parkin	—
**PHKG1**	Phosphorylase kinase, gamma 1 (muscle)	—
**PIN1**	Protein (peptidylprolyl cis/trans isomerase) NIMA-interacting 1	—
**PKN1**	Protein kinase N1	—
**PPP2CA**	Protein phosphatase 2 (formerly 2A), catalytic subunit, alpha isoform	—
**PPP2CB**	Protein phosphatase 2 (formerly 2A), catalytic subunit, beta isoform	—
**PPP2R5A**	Protein phosphatase 2, regulatory subunit B′, alpha isoform	—
**PPP5C**	Protein phosphatase 5, catalytic subunit	—
**PRKCD**	Protein kinase C, delta	—
**PSEN1**	Presenilin 1 (Alzheimer disease 3)	—
**RPS6KA3**	Ribosomal protein S6 kinase, 90 kDa, polypeptide 3	—
**RPS6KB1**	Ribosomal protein S6 kinase, 70 kDa, polypeptide 1	—
**S100B**	S100 calcium binding protein B	—
**SNCA**	Synuclein, alpha (non-A4 component of amyloid precursor)	—
**SPTB**	Spectrin, beta, erythrocytic (includes spherocytosis, clinical type I)	—
**STAU1**	Staufen, RNA binding protein, homolog 1 (Drosophila)	—
**STUB1**	STIP1 homology and U-box containing protein 1	—
**STXBP1**	Syntaxin binding protein 1	—
**TUBA4A**	Tubulin, alpha 4a	—
**TUBB**	Tubulin, beta	—
**UBC**	Ubiquitin C	Virion component
**YWHAB**	Tyrosine 3-monooxygenase/tryptophan 5-monooxygenase activation protein, beta polypeptide	—
**YWHAZ**	Tyrosine 3-monooxygenase/tryptophan 5-monooxygenase activation protein, zeta polypeptide	Virion component
